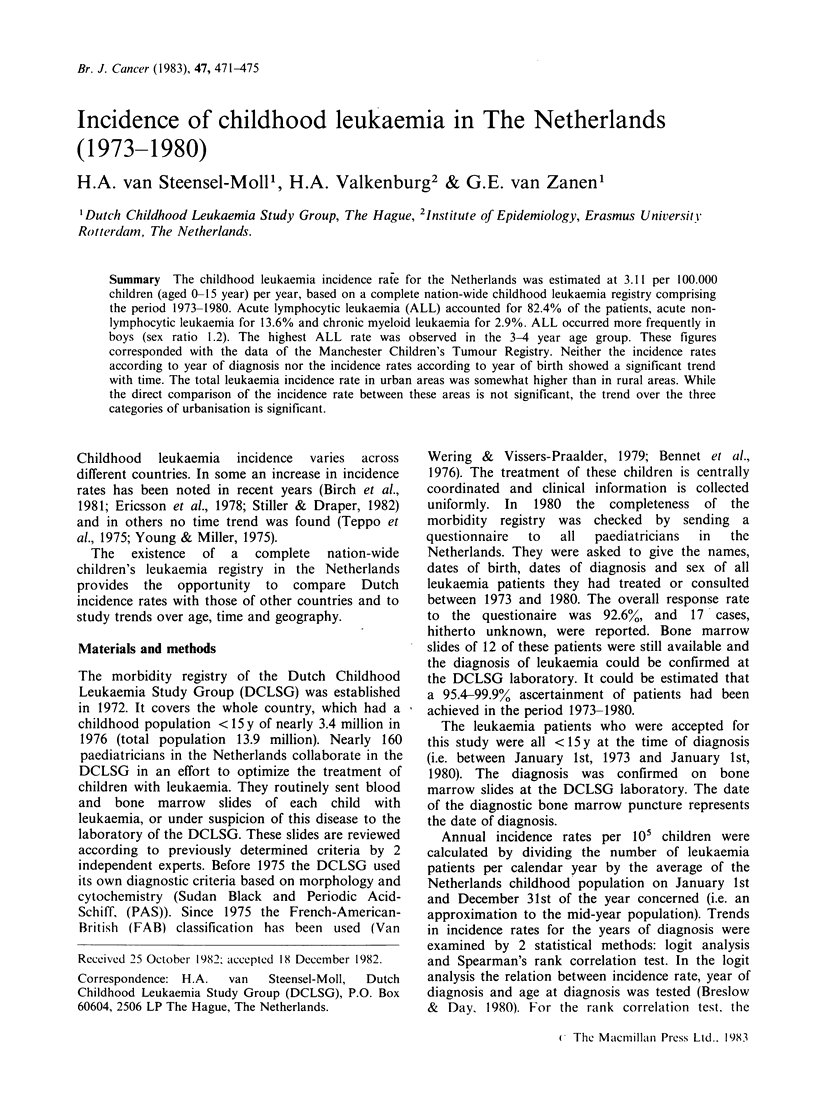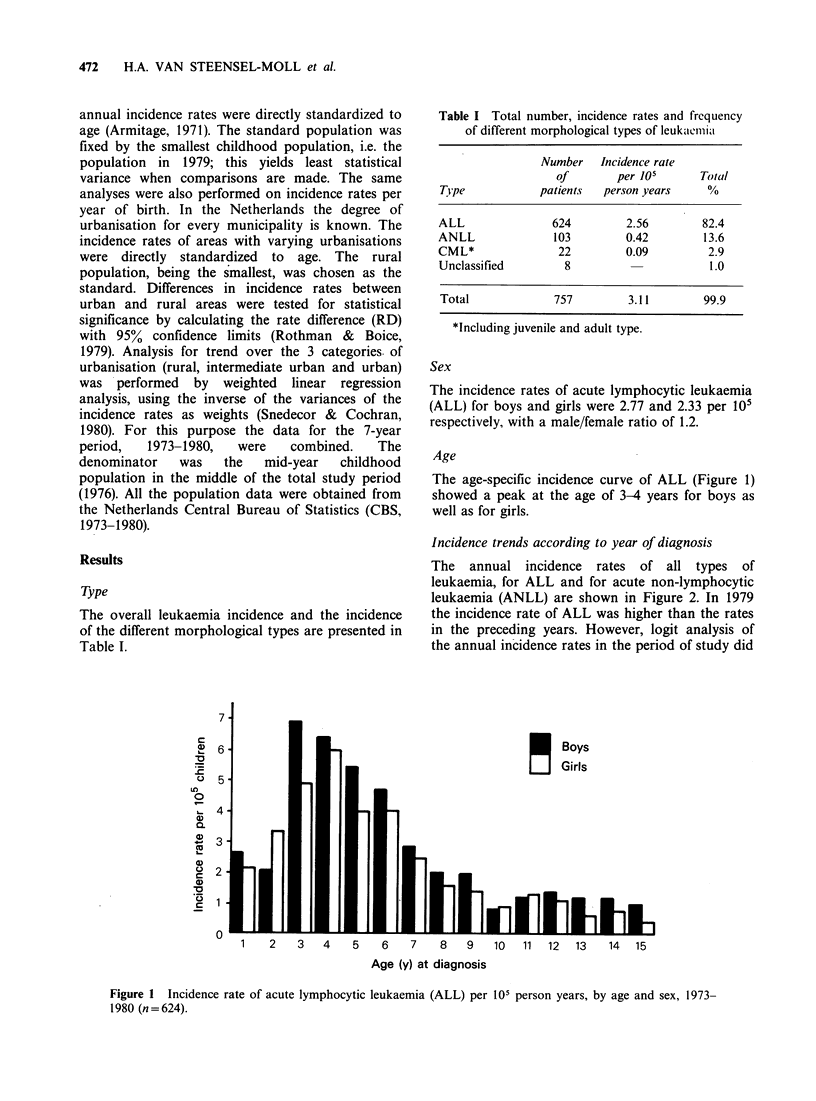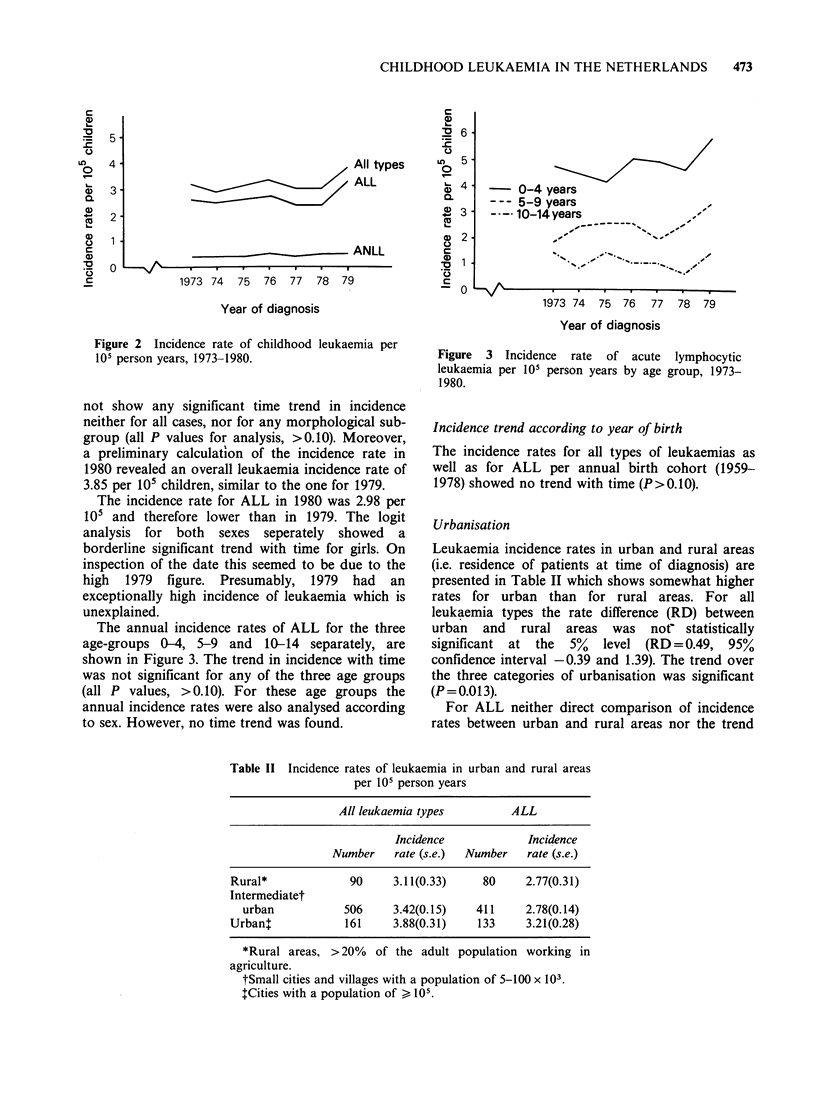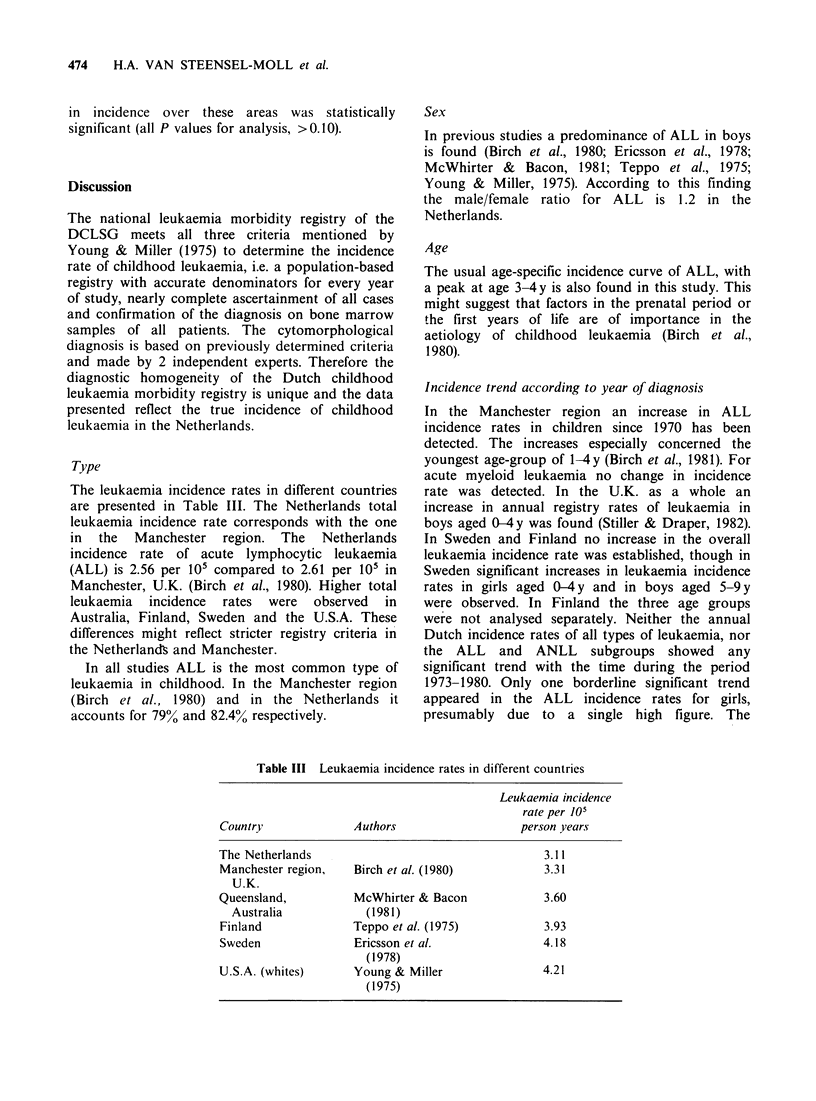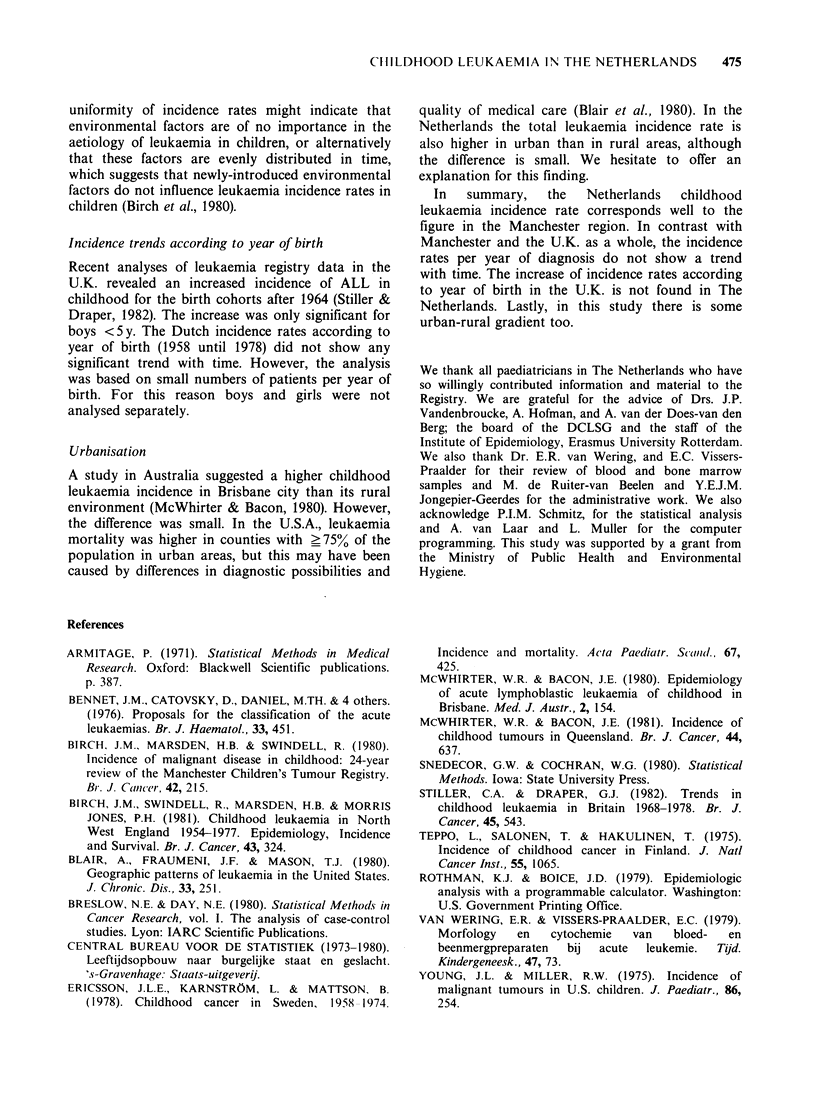# Incidence of childhood leukaemia in The Netherlands (1973-1980).

**DOI:** 10.1038/bjc.1983.76

**Published:** 1983-04

**Authors:** H. A. van Steensel-Moll, H. A. Valkenburg, G. E. van Zanen

## Abstract

The childhood leukaemia incidence rate for the Netherlands was estimated at 3.11 per 100.000 children (aged 0-15 year) per year, based on a complete nation-wide childhood leukaemia registry comprising the period 1973-1980. Acute lymphocytic leukaemia (ALL) accounted for 82.4% of the patients, acute non-lymphocytic leukaemia for 13.6% and chronic myeloid leukaemia for 2.9%. ALL occurred more frequently in boys (sex ratio 1.2). The highest ALL rate was observed in the 3-4 year age group. These figures corresponded with the data of the Manchester Children's Tumour Registry. Neither the incidence rates according to year of diagnosis nor the incidence rates according to year of birth showed a significant trend with time. The total leukaemia incidence rate in urban areas was somewhat higher than in rural areas. While the direct comparison of the incidence rate between these areas is not significant, the trend over the three categories of urbanisation is significant.


Br. J. Cancer (1983), 47, 471–475

### Incidence of childhood leukaemia in The Netherlands (1973–1980)

H.A. van Steensel-Moll[1], H.A. Valkenburg[2] & G.E. van Zanen[1]

[1]*Dutch Childhood Leukaemia Study Group, The Hague,* [2]*Institute of Epidemiology, Erasmus University Rotterdam, The Netherlands.*

**Summary** The childhood leukaemia incidence rate for the Netherlands was estimated at 3.11 per 100.000 children (aged 0–15 year) per year, based on a complete nation-wide childhood leukaemia registry comprising the period 1973–1980. Acute lymphocytic leukaemia (ALL) accounted for 82.4% of the patients, acute non-lymphocytic leukaemia for 13.6% and chronic myeloid leukaemia for 2.9%. ALL occurred more frequently in boys (sex ratio 1.2). The highest ALL rate was observed in the 3–4 year age group. These figures corresponded with the data of the Manchester Children's Tumour Registry. Neither the incidence rates according to year of diagnosis nor the incidence rates according to year of birth showed a significant trend with time. The total leukaemia incidence rate in urban areas was somewhat higher than in rural areas. While the direct comparison of the incidence rate between these areas is not significant, the trend over the three categories of urbanisation is significant.

Childhood leukaemia incidence varies across different countries. In some an increase in incidence rates has been noted in recent years (Birch *et al.*, 1981; Ericsson *et al.*, 1978; Stiller & Draper, 1982) and in others no time trend was found (Teppo *et al.*, 1975; Young & Miller, 1975).

The existence of a complete nation-wide children's leukaemia registry in the Netherlands provides the opportunity to compare Dutch incidence rates with those of other countries and to study trends over age, time and geography.

### Materials and methods

The morbidity registry of the Dutch Childhood Leukaemia Study Group (DCLSG) was established in 1972. It covers the whole country, which had a childhood population <15 y of nearly 3.4 million in 1976 (total population 13.9 million). Nearly 160 paediatricians in the Netherlands collaborate in the DCLSG in an effort to optimize the treatment of children with leukaemia. They routinely sent blood and bone marrow slides of each child with leukaemia, or under suspicion of this disease to the laboratory of the DCLSG. These slides are reviewed according to previously determined criteria by 2 independent experts. Before 1975 the DCLSG used its own diagnostic criteria based on morphology and cytochemistry (Sudan Black and Periodic Acid-Schiff, (PAS)). Since 1975 the French-American-British (FAB) classification has been used (Van Wering & Vissers-Praalder, 1979; Bennet *et al.*, 1976). The treatment of these children is centrally coordinated and clinical information is collected uniformly. In 1980 the completeness of the morbidity registry was checked by sending a questionnaire to all paediatricians in the Netherlands. They were asked to give the names, dates of birth, dates of diagnosis and sex of all leukaemia patients they had treated or consulted between 1973 and 1980. The overall response rate to the questionaire was 92.6%, and 17 cases, hitherto unknown, were reported. Bone marrow slides of 12 of these patients were still available and the diagnosis of leukaemia could be confirmed at the DCLSG laboratory. It could be estimated that a 95.4–99.9% ascertainment of patients had been achieved in the period 1973–1980.

The leukaemia patients who were accepted for this study were all <15 y at the time of diagnosis (i.e. between January 1st, 1973 and January 1st, 1980). The diagnosis was confirmed on bone marrow slides at the DCLSG laboratory. The date of the diagnostic bone marrow puncture represents the date of diagnosis.

Annual incidence rates per $10^5$ children were calculated by dividing the number of leukaemia patients per calendar year by the average of the Netherlands childhood population on January 1st and December 31st of the year concerned (i.e. an approximation to the mid-year population). Trends in incidence rates for the years of diagnosis were examined by 2 statistical methods: logit analysis and Spearman's rank correlation test. In the logit analysis the relation between incidence rate, year of diagnosis and age at diagnosis was tested (Breslow & Day, 1980). For the rank correlation test, the

Received 25 October 1982; accepted 18 December 1982.

Correspondence: H.A. van Steensel-Moll, Dutch Childhood Leukaemia Study Group (DCLSG), P.O. Box 60604, 2506 LP The Hague, The Netherlands.

© The Macmillan Press Ltd., 1983

annual incidence rates were directly standardized to age (Armitage, 1971). The standard population was fixed by the smallest childhood population, i.e. the population in 1979; this yields least statistical variance when comparisons are made. The same analyses were also performed on incidence rates per year of birth. In the Netherlands the degree of urbanisation for every municipality is known. The incidence rates of areas with varying urbanisations were directly standardized to age. The rural population, being the smallest, was chosen as the standard. Differences in incidence rates between urban and rural areas were tested for statistical significance by calculating the rate difference (RD) with 95% confidence limits (Rothman & Boice, 1979). Analysis for trend over the 3 categories of urbanisation (rural, intermediate urban and urban) was performed by weighted linear regression analysis, using the inverse of the variances of the incidence rates as weights (Snedecor & Cochran, 1980). For this purpose the data for the 7-year period, 1973–1980, were combined. The denominator was the mid-year childhood population in the middle of the total study period (1976). All the population data were obtained from the Netherlands Central Bureau of Statistics (CBS, 1973–1980).

### Results

### *Type*

The overall leukaemia incidence and the incidence of the different morphological types are presented in Table I.

**Table I** Total number, incidence rates and frequency of different morphological types of leukaemia

| *Type* | *Number of patients* | *Incidence rate per $10^5$ person years* | *Total %* |
|---|---|---|---|
| ALL | 624 | 2.56 | 82.4 |
| ANLL | 103 | 0.42 | 13.6 |
| CML* | 22 | 0.09 | 2.9 |
| Unclassified | 8 | — | 1.0 |
| Total | 757 | 3.11 | 99.9 |

*Including juvenile and adult type.

### *Sex*

The incidence rates of acute lymphocytic leukaemia (ALL) for boys and girls were 2.77 and 2.33 per $10^5$ respectively, with a male/female ratio of 1.2.

### *Age*

The age-specific incidence curve of ALL (Figure 1) showed a peak at the age of 3–4 years for boys as well as for girls.

### *Incidence trends according to year of diagnosis*

The annual incidence rates of all types of leukaemia, for ALL and for acute non-lymphocytic leukaemia (ANLL) are shown in Figure 2. In 1979 the incidence rate of ALL was higher than the rates in the preceding years. However, logit analysis of the annual incidence rates in the period of study did

**Figure 1** Incidence rate of acute lymphocytic leukaemia (ALL) per $10^5$ person years, by age and sex, 1973–1980 ($n = 624$).

**Figure 2** Incidence rate of childhood leukaemia per $10^5$ person years, 1973–1980.

**Figure 3** Incidence rate of acute lymphocytic leukaemia per $10^5$ person years by age group, 1973–1980.

not show any significant time trend in incidence neither for all cases, nor for any morphological subgroup (all $P$ values for analysis, $>0.10$). Moreover, a preliminary calculation of the incidence rate in 1980 revealed an overall leukaemia incidence rate of 3.85 per $10^5$ children, similar to the one for 1979.

The incidence rate for ALL in 1980 was 2.98 per $10^5$ and therefore lower than in 1979. The logit analysis for both sexes seperately showed a borderline significant trend with time for girls. On inspection of the date this seemed to be due to the high 1979 figure. Presumably, 1979 had an exceptionally high incidence of leukaemia which is unexplained.

The annual incidence rates of ALL for the three age-groups 0–4, 5–9 and 10–14 separately, are shown in Figure 3. The trend in incidence with time was not significant for any of the three age groups (all $P$ values, $>0.10$). For these age groups the annual incidence rates were also analysed according to sex. However, no time trend was found.

### *Incidence trend according to year of birth*

The incidence rates for all types of leukaemias as well as for ALL per annual birth cohort (1959–1978) showed no trend with time ($P>0.10$).

### *Urbanisation*

Leukaemia incidence rates in urban and rural areas (i.e. residence of patients at time of diagnosis) are presented in Table II which shows somewhat higher rates for urban than for rural areas. For all leukaemia types the rate difference (RD) between urban and rural areas was not statistically significant at the 5% level (RD=0.49, 95% confidence interval −0.39 and 1.39). The trend over the three categories of urbanisation was significant ($P=0.013$).

For ALL neither direct comparison of incidence rates between urban and rural areas nor the trend

**Table II** Incidence rates of leukaemia in urban and rural areas per $10^5$ person years

| | *All leukaemia types* | | *ALL* | |
|---|---|---|---|---|
| | *Number* | *Incidence rate (s.e.)* | *Number* | *Incidence rate (s.e.)* |
| Rural* | 90 | 3.11(0.33) | 80 | 2.77(0.31) |
| Intermediate† urban | 506 | 3.42(0.15) | 411 | 2.78(0.14) |
| Urban‡ | 161 | 3.88(0.31) | 133 | 3.21(0.28) |

*Rural areas, >20% of the adult population working in agriculture.

†Small cities and villages with a population of $5–100 \times 10^3$.

‡Cities with a population of $\geqslant 10^5$.

in incidence over these areas was statistically significant (all *P* values for analysis, >0.10).

### Discussion

The national leukaemia morbidity registry of the DCLSG meets all three criteria mentioned by Young & Miller (1975) to determine the incidence rate of childhood leukaemia, i.e. a population-based registry with accurate denominators for every year of study, nearly complete ascertainment of all cases and confirmation of the diagnosis on bone marrow samples of all patients. The cytomorphological diagnosis is based on previously determined criteria and made by 2 independent experts. Therefore the diagnostic homogeneity of the Dutch childhood leukaemia morbidity registry is unique and the data presented reflect the true incidence of childhood leukaemia in the Netherlands.

### *Type*

The leukaemia incidence rates in different countries are presented in Table III. The Netherlands total leukaemia incidence rate corresponds with the one in the Manchester region. The Netherlands incidence rate of acute lymphocytic leukaemia (ALL) is 2.56 per $10^5$ compared to 2.61 per $10^5$ in Manchester, U.K. (Birch *et al.*, 1980). Higher total leukaemia incidence rates were observed in Australia, Finland, Sweden and the U.S.A. These differences might reflect stricter registry criteria in the Netherlands and Manchester.

In all studies ALL is the most common type of leukaemia in childhood. In the Manchester region (Birch *et al.*, 1980) and in the Netherlands it accounts for 79% and 82.4% respectively.

### *Sex*

In previous studies a predominance of ALL in boys is found (Birch *et al.*, 1980; Ericsson *et al.*, 1978; McWhirter & Bacon, 1981; Teppo *et al.*, 1975; Young & Miller, 1975). According to this finding the male/female ratio for ALL is 1.2 in the Netherlands.

### *Age*

The usual age-specific incidence curve of ALL, with a peak at age 3–4 y is also found in this study. This might suggest that factors in the prenatal period or the first years of life are of importance in the aetiology of childhood leukaemia (Birch *et al.*, 1980).

### *Incidence trend according to year of diagnosis*

In the Manchester region an increase in ALL incidence rates in children since 1970 has been detected. The increases especially concerned the youngest age-group of 1–4 y (Birch *et al.*, 1981). For acute myeloid leukaemia no change in incidence rate was detected. In the U.K. as a whole an increase in annual registry rates of leukaemia in boys aged 0–4 y was found (Stiller & Draper, 1982). In Sweden and Finland no increase in the overall leukaemia incidence rate was established, though in Sweden significant increases in leukaemia incidence rates in girls aged 0–4 y and in boys aged 5–9 y were observed. In Finland the three age groups were not analysed separately. Neither the annual Dutch incidence rates of all types of leukaemia, nor the ALL and ANLL subgroups showed any significant trend with the time during the period 1973–1980. Only one borderline significant trend appeared in the ALL incidence rates for girls, presumably due to a single high figure. The

**Table III** Leukaemia incidence rates in different countries

| *Country* | *Authors* | *Leukaemia incidence rate per $10^5$ person years* |
|---|---|---|
| The Netherlands | | 3.11 |
| Manchester region, U.K. | Birch *et al.* (1980) | 3.31 |
| Queensland, Australia | McWhirter & Bacon (1981) | 3.60 |
| Finland | Teppo *et al.* (1975) | 3.93 |
| Sweden | Ericsson *et al.* (1978) | 4.18 |
| U.S.A. (whites) | Young & Miller (1975) | 4.21 |

uniformity of incidence rates might indicate that environmental factors are of no importance in the aetiology of leukaemia in children, or alternatively that these factors are evenly distributed in time, which suggests that newly-introduced environmental factors do not influence leukaemia incidence rates in children (Birch *et al.*, 1980).

### *Incidence trends according to year of birth*

Recent analyses of leukaemia registry data in the U.K. revealed an increased incidence of ALL in childhood for the birth cohorts after 1964 (Stiller & Draper, 1982). The increase was only significant for boys $<5$ y. The Dutch incidence rates according to year of birth (1958 until 1978) did not show any significant trend with time. However, the analysis was based on small numbers of patients per year of birth. For this reason boys and girls were not analysed separately.

### *Urbanisation*

A study in Australia suggested a higher childhood leukaemia incidence in Brisbane city than its rural environment (McWhirter & Bacon, 1980). However, the difference was small. In the U.S.A., leukaemia mortality was higher in counties with $\geq 75\%$ of the population in urban areas, but this may have been caused by differences in diagnostic possibilities and quality of medical care (Blair *et al.*, 1980). In the Netherlands the total leukaemia incidence rate is also higher in urban than in rural areas, although the difference is small. We hesitate to offer an explanation for this finding.

In summary, the Netherlands childhood leukaemia incidence rate corresponds well to the figure in the Manchester region. In contrast with Manchester and the U.K. as a whole, the incidence rates per year of diagnosis do not show a trend with time. The increase of incidence rates according to year of birth in the U.K. is not found in The Netherlands. Lastly, in this study there is some urban-rural gradient too.

We thank all paediatricians in The Netherlands who have so willingly contributed information and material to the Registry. We are grateful for the advice of Drs. J.P. Vandenbroucke, A. Hofman, and A. van der Does-van den Berg; the board of the DCLSG and the staff of the Institute of Epidemiology, Erasmus University Rotterdam. We also thank Dr. E.R. van Wering, and E.C. Vissers-Praalder for their review of blood and bone marrow samples and M. de Ruiter-van Beelen and Y.E.J.M. Jongepier-Geerdes for the administrative work. We also acknowledge P.I.M. Schmitz, for the statistical analysis and A. van Laar and L. Muller for the computer programming. This study was supported by a grant from the Ministry of Public Health and Environmental Hygiene.

**References**

ARMITAGE, P. (1971). *Statistical Methods in Medical Research*. Oxford: Blackwell Scientific publications. p. 387.

BENNET, J.M., CATOVSKY, D., DANIEL, M.TH. & 4 others. (1976). Proposals for the classification of the acute leukaemias. *Br. J. Haematol.*, **33**, 451.

BIRCH, J.M., MARSDEN, H.B. & SWINDELL, R. (1980). Incidence of malignant disease in childhood: 24-year review of the Manchester Children's Tumour Registry. *Br. J. Cancer*, **42**, 215.

BIRCH, J.M., SWINDELL, R., MARSDEN, H.B. & MORRIS JONES, P.H. (1981). Childhood leukaemia in North West England 1954–1977. Epidemiology, Incidence and Survival. *Br. J. Cancer*, **43**, 324.

BLAIR, A., FRAUMENI, J.F. & MASON, T.J. (1980). Geographic patterns of leukaemia in the United States. *J. Chronic. Dis.*, **33**, 251.

BRESLOW, N.E. & DAY, N.E. (1980). *Statistical Methods in Cancer Research*, vol. I. The analysis of case-control studies. Lyon: IARC Scientific Publications.

CENTRAL BUREAU VOOR DE STATISTIEK (1973–1980). Leeftijdsopbouw naar burgelijke staat en geslacht. *'s-Gravenhage: Staats-uitgeverij.*

ERICSSON, J.L.E., KARNSTRÖM, L. & MATTSON, B. (1978). Childhood cancer in Sweden, 1958–1974. Incidence and mortality. *Acta Paediatr. Scand.*, **67**, 425.

MCWHIRTER, W.R. & BACON, J.E. (1980). Epidemiology of acute lymphoblastic leukaemia of childhood in Brisbane. *Med. J. Austr.*, **2**, 154.

MCWHIRTER, W.R. & BACON, J.E. (1981). Incidence of childhood tumours in Queensland. *Br. J. Cancer*, **44**, 637.

SNEDECOR, G.W. & COCHRAN, W.G. (1980). *Statistical Methods*. Iowa: State University Press.

STILLER, C.A. & DRAPER, G.J. (1982). Trends in childhood leukaemia in Britain 1968–1978. *Br. J. Cancer*, **45**, 543.

TEPPO, L., SALONEN, T. & HAKULINEN, T. (1975). Incidence of childhood cancer in Finland. *J. Natl Cancer Inst.*, **55**, 1065.

ROTHMAN, K.J. & BOICE, J.D. (1979). Epidemiologic analysis with a programmable calculator. Washington: U.S. Government Printing Office.

VAN WERING, E.R. & VISSERS-PRAALDER, E.C. (1979). Morfology en cytochemie van bloed- en beenmergpreparaten bij acute leukemie. *Tijd. Kindergeneesk.*, **47**, 73.

YOUNG, J.L. & MILLER, R.W. (1975). Incidence of malignant tumours in U.S. children. *J. Paediatr.*, **86**, 254.